# Antibacterial Activity of Emulsified Pomelo (*Citrus grandis* Osbeck) Peel Oil and Water-Soluble Chitosan on *Staphylococcus aureus* and *Escherichia coli*

**DOI:** 10.3390/molecules23040840

**Published:** 2018-04-06

**Authors:** Guan-Wen Chen, Yu-Hsin Lin, Chia-Hua Lin, Hsiao-Chin Jen

**Affiliations:** 1 Department of Food Science, National Taiwan Ocean University, No. 2 Pei-Ning Rd., Keelung 202, Taiwan; chengw@mail.ntou.edu.tw; 2 Department of Food Technology and Marketing, Taipei University of Marine Technology, No. 212, Sec. 9, Yan Ping N. Rd., Taipei 111, Taiwan; 3 Department of Biotechnology, National Formosa University, No. 64, Wunhua Rd, Yunlin 632, Taiwan; vicchlin@nfu.edu.tw; 4 Department of Health Promotion and Gerontological Care, Taipei University of Marine Technology, No. 212, Sec. 9, Yan Ping N. Rd., Taipei 111, Taiwan; f1216@mail.tcmt.edu.tw

**Keywords:** essential oil, pomelo, antibacterial activity, emulsified essential oil, water-soluble chitosan

## Abstract

This study utilized pomelo steam distillation to isolate pomelo peel essential oil. The constituents were then analyzed through gas chromatography-mass spectrometry (GC-MS), and the antibacterial activity of the essential oil emulsions at different homogenizer speed conditions and concentrations of water-soluble chitosan (degree of acetylation, DA = 54.8%) against *S. aureus* and *E. coli* was examined. Analysis of the essential oil composition identified a total of 33 compounds with the main constituent, limonene accounting for 87.5% (940.07 mg/g) of the total. The pomelo peel oil was emulsified through homogenization at 24,000 rpm, resulting in a minimal inhibitory concentration (MIC) for *E. coli* that was 1.9 times lower than that of the essential oil without homogenization. In addition, a mixture of 0.4% essential oil emulsion and 0.03% water-soluble chitosan had the strongest synergetic antibacterial effect on *S. aureus* and *E. coli* at pH 7.4. In comparison with chitosan alone, the MIC value of this mixture was significantly 2.4 and 2.5 times lower. Hence, this study suggests using a mixture of emulsified pomelo peel oil and water-soluble chitosan to develop a novel natural food preservative, and that the processability of food, as well as the economic value of the byproducts of the Taiwan Matou pomelo and chitosan, could be increased.

## 1. Introduction

Food safety is closely related to public health, a topic that has gained increasing attention. According to current knowledge, ingesting food contaminated by pathogenic microorganisms, such as *Staphylococcus aureus* and *Escherichia coli*, may increase health risks. To prevent food from being contaminated by microorganisms, chemosynthetic preservatives are prevalent in the food industry [1]. However, as consumers have become more aware of food safety issues, the demand for food free of chemical preservatives has gradually grown. Therefore, the development of a natural and safe antimicrobial agent has become more important. 

*Citrus grandis* Osbeck, also known as pomelo, is a popular seasonal fruit in Taiwan. It is regularly consumed in Asian countries as a whole fruit, juice, or preserved snack [2]. The annual yield of pomelo in Taiwan is 70,000 t [3]. The pomelo is a popular fruit for Chinese festivals; however, they are sometimes overproduced, have slow sales and are discarded after festivals. This fruit, and its processed products, produce considerable waste or byproducts during eating or processing, such as peels, seeds, and pulp, which account for approximately 50% of the original fruit weight [4]. These byproducts could be a valuable source for functional food ingredients, such as flavonoids, dietary fibers, and essential oils [5]. Essential oil is the most important byproduct of citrus processing and is usually obtained from the peels. Essential oils are mixtures of aromatic volatile compounds produced by the secondary metabolism of plants and they can be used as natural antimicrobials [6]. Several reports have addressed the antimicrobial activity of essential oils, as well as the use of essential oils as antimicrobial agents in food products. However, many potential technological challenges remain associated with incorporating essential oils into food products, due to their low water solubility, poor chemical stability, and volatile nature [7]. In recent years, many studies have used emulsification or nanotechnology to improve the characteristics of essential oils to enhance its usability and stability, in order to enhance its applicability to food [8,9]. However, research into the antimicrobial activity of emulsified citrus essential oil under different homogenization conditions is limited. 

Chitosan, being produced from a major waste product of the marine food processing industry, is widely available. It is a biodegradable, nontoxic biopolymer of glucosamine and *N*-acetylglucosamine that is commercially produced by deacetylating chitin, the main structural component of crustacean shells. Chitosan exhibits a wide array of potential biological activities, such as antitumor, immunostimulatory, antibacterial, and antifungal activities [10,11]. The antimicrobial activity of chitosan against a variety of bacteria and fungi is known to be due to its polycationic nature [11,12]. However, its antibacterial activity is limited at a pH higher than 6.5, when chitosan starts to lose its positive charge and become insoluble in water [13,14]. Thus, many researchers have focused on the preparation of chitosan and chitosan derivatives that are soluble in water over a wider pH range through enzymatic and acid hydrolysis. In addition, chitosan is widely used as an antimicrobial agent when blended with other natural polymers [10,15]. However, research into the antimicrobial activity of water-soluble chitosan with about 50.0% degree of acetylation blended with citrus essential oil emulsion is limited. 

This study used Taiwan Matou pomelo peel as the raw material and performed industrially safe and nontoxic steam distillation to obtain the essential oil. The main composition was analyzed, and the effect of essential oil emulsions after different homogenization conditions was investigated, Then, the essential oil was blended with different concentrations of water-soluble chitosan with about 50.0% degree of acetylation and the antimicrobial activity was examined. Common foodborne pathogenic bacteria, the gram-negative *E. coli* and the gram-positive *S. aureus*, were used as antimicrobial index strains. An emulsion mixture of pomelo peel essential oil and water-soluble chitosan is expected to be developed in the future, to be used in the food and catering industries as a natural water-soluble germicide or food preservative. This will increase the economic value of Taiwan Matou pomelo peel and chitin wastes.

## 2. Results and Discussion

### 2.1. Analysis of Pomelo Peel Essential Oil Composition

Essential oil was obtained from the fresh peel of the Taiwan Matou cultivar of the pomelo (*Citrus grandis* [L.] Osbeck cv. Matou Wentan) through steam distillation. The essential oil composition was analyzed through GC-MS, and 33 compounds were identified. According to gas chromatography (GC) quantitative analysis, the main constituents in descending order of content were limonene (87.5%, 940.07 mg/g), myrcene (3.1%, 23.65 mg/g), β-pinene (2.7%, 13.53 mg/g), α-pinene (6.0%, 4.66 mg/g), *cis*-linalool oxide (f) (0.6%, 3.82 mg/g), and linalool (0.4%, 2.48 mg/g) (Table 1). This is similar to the findings of previous investigations into pomelo peel oils, which reported that limonene (93.9%), myrcene (1.9%), β-pinene (1.1%), and α-pinene (0.5%) were the chief components [16]. Previous studies have shown that limonene (62.0–95.4%) was the principal constituent of pomelo peel oils [16,17]. Differences in the limonene content of the essential oil of pomelo peel may be due to differences in the growth environment, provenance, isolation conditions, and the part of the plant where the oil was obtained [16,17].

### 2.2. Antibacterial Activity of Essential Oil

The solutions of essential oil at different concentrations with or without homogenization were used for antibacterial activity tests involving *S. aureus* and *E. coli* (Table 2). 

Tween 20 was utilized as a surfactant to facilitate emulsion formation and stability, and 1% Tween 20 aqueous solution without essential oil served as the control group. The antibacterial effect increased with the essential oil concentration. The antibacterial effect of the unhomogenized essential oil on *S. aureus* and *E. coli* was the strongest when the concentration was 0.4% (4 μL/mL); the total bacterial counts of *S. aureus* (7.97 ± 0.23 CFU/mL) and *E. coli* (8.18 ± 0.13 CFU/mL) were lower than those of the control group by 1.03 and 0.85 log CFU/mL, respectively. According to the results, the pomelo peel essential oil had a greater inhibitory effect on *S. aureus* than on *E. coli*. This result is consistent with previous findings on antibacterial citrus essential oils and suggests that the essential oil and its compounds have a stronger inhibitory effect on gram-positive bacteria than on gram-negative bacteria [6,18]. In addition, the antibacterial effects observed at different homogenizer speeds and the same essential oil concentration indicate that the homogenizer speed has no significant effect (*p* < 0.05) on the antibacterial activity of *S. aureus*, though the antibacterial effect increases with the homogenizer speed. When the essential oil concentration was increased to 0.4% (*v*/*v*) and the homogenizer speed was 24,000 rpm, the total bacterial counts of *E. coli* were lower than those of the unhomogenized group and the 13,500 rpm group by 0.46 and 0.33 log CFU/mL, respectively (Table 2). The MIC values for *S. aureus* and *E. coli* were calculated from the regression curve between various essential oil concentrations at different homogenizer speeds (0, 13,500, and 24,000 rpm) and the residual bacterial count. The MIC values of the unhomogenized group and 24,000 rpm group for *S. aureus* were 0.29% (2.9 μL/mL) and 0.27% (2.7 μL/mL), respectively. For *E. coli*, the MIC value of the essential oil emulsion homogenized at 24,000 rpm was 0.25% (2.5 μL/mL), whereas that of the unhomogenized group was 0.49% (4.9 μL/mL) (Table 5). According to the results of this study, homogenization at 24,000 rpm markedly enhanced the inhibitory effect of the essential oil emulsion on *E. coli*, with the MIC being 1.9 times lower than that of the essential oil without homogenization. In a study by Inouye et al., the MIC value of citron oil, and its key compound limonene, for *S. aureus* and *E. coli* was >0.32% (*w*/*v*) [6]. Additionally, Moghimi et al. found that the MIC value of a nanoemulsion of Thymus daenensis essential oil against *E. coli* was 10 times higher than that of pure essential oil, with the MIC values being 0.4 mg/mL and 4.0 mg/mL, respectively [9]. Emulsification enhances the antibacterial effect of essential oil on *E. coli*, which may be related to the speed of homogenization. The higher the homogenizer speed is, the more complete the emulsification; the size of the oil droplets decreases, the stability of the oil droplets is enhanced, and the oil droplets are uniformly dispersed in the overall solution system, which enhances the ability of the oil to permeate the hydrophilic pericellular membrane of the gram-negative bacterium *E. coli* and thus improves antibacterial activity [8,9]. The results of this study are in agreement with previous studies, indicating that the antibacterial effects are greatly improved through the conversion of flavor or essential oils (e.g., oregano oil and d-limonene) into nanoemulsions [8,19].

### 2.3. Antibacterial Activity of Water-Soluble Chitosan

Water-soluble chitosan (DA = 54.80%) was prepared by using acetic anhydride acetylation, and the antibacterial effects of water-soluble chitosan (pH 7.4) at different concentrations (0.01%, 0.03%, 0.05%, and 0.1%) on *S. aureus* and *E. coli* were measured (Table 3). 

The results showed that the antibacterial effect of water-soluble chitosan on *S. aureus* and *E. coli* increased with the concentration of chitosan. At a concentration of 0.1%, water-soluble chitosan exerted the strongest antibacterial effect on *S. aureus* and *E. coli*. The total bacterial counts of *S. aureus* and *E. coli* were lower than those of the control group by 0.96 and 1.07 log CFU/mL, respectively (Table 3). The findings of this study are similar to those reported by Chen, et al., who used water-soluble chitosan with a DA of 50%; when the concentration was 1,000 ppm, the bacterial count of *S. aureus* was reduced by about 1.0 log CFU/mL after 12 h of cultivation [13]. In addition, Omura et al. reported that once the degree of *N*-acetylation reached approximately 55%, chitosan had a good inhibitory effect on *S. aureus* and *E. coli*, with an MIC value of >0.1% (*w*/*v*) (equivalent to >1000 ppm) [14]. The antibacterial effectiveness of chitosan has been demonstrated against both gram-positive and gram-negative bacteria, with an MIC range of 100 to 2000 ppm [20]. In the current study, the MIC values for *S. aureus* and *E. coli* were calculated from the regression curve between the concentration of water-soluble chitosan and residual bacterial count to be 0.046% and 0.086%, respectively (Table 5). 

### 2.4. Antibacterial Activity of Essential Oil Emulsion and Water-Soluble Chitosan

The water-soluble chitosan at different concentrations was mixed with a 0.4% essential oil emulsion (homogenized at 24,000 rpm), and the antibacterial effects of the combination of the essential oil emulsion and water-soluble chitosan on *S. aureus* and *E. coli* were measured (Table 4).

The antibacterial effects of chitosan at concentrations of 0, 0.01%, 0.03%, 0.05%, and 0.1% on *S. aureus* were lower than those of the control group by 1.06, 1.97, 2.40, 2.34, and 2.17 log CFU/mL, respectively; the effects on *E. coli* were lower by 1.25, 1.79, 2.26, 2.55, and 2.08 log CFU/mL, respectively (Table 4). The results showed that the combination of the essential oil emulsion and water-soluble chitosan can significantly enhance the antibacterial effect on *S. aureus* and *E. coli*. In addition, in comparison with the antibacterial effect of only water-soluble chitosan (no emulsified essential oil) on *S. aureus*, the MIC value of the combination of the essential oil emulsion and water-soluble chitosan was 2.4 times higher, with the MIC values being 0.046% and 0.019%, respectively (Table 5). Moreover, the MIC value of the combination of the essential oil emulsion and water-soluble chitosan on *E. coli* was 2.5 times higher, with the MIC value being 0.034%, compared with 0.086% of chitosan alone (Table 5). This result may be related to a synergistic effect of water-soluble chitosan and emulsified pomelo peel oil on bacterial activity. This coincides with the results of Feyzioglu and Tornuk, who suggested that chitosan nanoparticles loaded with essential oil from summer savory provide significant reductions in bacteria (*E. coli* O157:H7, *Listeria monocytogenes*, and *S. aureus*) in comparison with chitosan nanoparticles alone [21]. However, in the current study, when the water-soluble chitosan concentration in the 0.4% essential oil emulsion was 0.1%, the antibacterial effect on *S. aureus* and *E. coli* was lower than that in the 0.03% and 0.05% groups. However, the differences were not significant (Table 4), which may be because the high concentration of chitosan reduced the contact between the hydrophobic essential oil compound and the cell wall of the bacteria, obstructing the channels for the essential oil compound to enter the bacteria. Thus, the essential oil compound could not permeate the bacterial cells, resulting in lower bacteriostatic activity against *S. aureus* and *E. coli* [22]. 

The 0.4% pomelo peel essential oil emulsion mixed with 0.03% water-soluble chitosan had a significant antibacterial effect on *S. aureus* and *E. coli* (*p* < 0.05) (Table 4). In addition, the MIC values of this mixture for *S. aureus* and *E. coli* were 0.019% and 0.034%, respectively. As such, the MIC value of this mixture for *S. aureus* was higher than that of the 0.01% water-soluble chitosan, but the value for *E. coli* was nearest to the 0.03% water-soluble chitosan. Therefore, we further analyzed the antibacterial activity and stability of this mixture at various pH values. 

### 2.5. Antibacterial Activity of the Combination of Essential Oil and Chitosan at Various pH Values

When the culture media at pH 5.5, 7.4, and 8.5 were mixed only with the 0.4% essential oil emulsion, the *S. aureus* inhibition rates were 81.20%, 90.04%, and 95.10%, respectively, suggesting that the pH 8.5 basic essential oil emulsion has the highest inhibitory effect on *S. aureus* (Table 6). 

This result is similar to the result of Hoque et al. [23]. Clove isolation exhibited higher antibacterial activity against *S. aureus* at pH 9.0, compared with pH 5.0 and 7.0. In addition, essential clove oil exhibited higher antibacterial activity against gram-positive bacteria (*L. monocytogenes*) at pH 9.0 than at pH 5.0 and 7.0 [23]. Regarding the effect of the pH value on the activity of essential oil, the pH value may influence charge modification on the cell membrane surface; thus, the charged constituents of the essential oil bind to the microorganism cell membrane, which may damage the cells [24]. However, the *E. coli* inhibition rates of the essential oil emulsion alone were 78.11%, 92.7%, and 71.29%, indicating that neutral (pH 7.4) essential oil emulsions have the highest inhibitory effect, followed by acidic emulsions. Generally, a low pH increases the hydrophobicity of the essential oil, and the resistance of bacteria to this essential oil and its constituents decreases with the pH value; thus, it is easier for essential oils to dissolve in the lipids of the bacterial cell membrane [25]. However, according to findings regarding citrus essential oils, the principal component limonene, which belongs to the family of cyclic monoterpene hydrocarbons, is believed to accumulate in the bacterial cell membrane, which results in the loss of membrane integrity and leads to dissipation of the proton motive force [26]. Previous studies on the inactivation of *E. coli* by limonene demonstrated that it targets the phospholipids of cytoplasmic membranes and the lipopolysaccharide fraction of the cell wall at pH 7.0 and β-sheet protein out of the membrane at pH 4.0, which results in sublethal injury in the outer and cytoplasmic membranes [27]. Furthermore, water-soluble chitosan at 0.03% and pH 5.5 had the highest inhibitory effect; the *S. aureus* inhibition rate was 85.47%, and the *E. coli* inhibition rate was 76.04% (Table 6). For water-soluble chitosan in TSB solutions at different pH values (5.5, 7.4, and 8.5), the particle surface charge values were 5.4, −3.8, and −3.2 mV, respectively (Table 6). Cationic (NH^3+^) forms of chitosan are present in acidic pH, while in neutral and basic pH, NH_2_ groups are present. The number of NH_2_ groups is related not only to chitosan concentration but also to the degree of acetylation, that is, it diminished when the degree of acetylation increased [28]. This result indicates that water-soluble chitosan is positively charged at pH 5.5; thus, the inhibitory effect on *S. aureus* and *E. coli* is significantly (*p* < 0.05) higher than that at pH 7.4 and 8.5. This finding is similar to that of Xie et al. [29]. For the mixture of the essential oil emulsion and water-soluble chitosan, the *S. aureus* inhibition rate (82.58%) of 0.03% chitosan with 0.4% essential oil at pH 5.5 was not significantly different, but in comparison with the 0.4% essential oil emulsion (90.04% and 95%) or the 0.03% water-soluble chitosan (65.26% and 61.83%) alone, the chitosan and oil at pH 7.4 and 8.5 had a synergistic antibacterial effect on *S. aureus*, with inhibition rates of 99.48% and 97.46%, respectively. Moreover, in comparison with the 0.4% essential oil emulsion (78.11%, 92.70%, and 71.29%) or the 0.03% water-soluble chitosan (76.04%, 62.61%, and 66.06%) groups, the chitosan and oil at pH 5.5, 7.4, and 8.5 had significant synergistic antibacterial effects on *E. coli*, and the values were 91.68%, 99.06%, and 95.06%, respectively (Table 6). Despite the differences between the gram-positive and gram-negative species, the mixture of 0.03% water-soluble chitosan and the 0.4% essential oil emulsion at pH 7.4 had the highest antibacterial effect on both *S. aureus* and *E. coli*. In comparison with the total bacterial count of the control group, the values were 2.40 and 2.26 log CFU/mL lower (Table 4). Additionally, in comparison with the 0.4% essential oil emulsion and 0.03% water-soluble chitosan individually, the effects of this mixture in reducing the total bacterial counts of *S. aureus* were 1.8 and 4.6 times higher, respectively, and for *E. coli* were 2.2 and 4.8 times higher, respectively (Table 3 and Table 4). The results showed that the mixture had significantly enhanced synergistic antibacterial effects on *S. aureus* and *E. coli*. The results of this study are similar to those of Feyziglu et al., who investigated the synergistic antibacterial activities of chitosan nanoparticles loaded with summer savory essential oil against *S. aureus* and *E. coli* O157:H7. However, the mixture’s exact mechanism of action remains unclear [21]. The essential oil emulsion can enhance antibacterial synergy with water-soluble chitosan in neutral solution. This is not attributable to a specific compound or mechanism, but due to the presence of various chemical compounds with different structures in the pomelo peel essential oil [21]. In fact, the essential oils affect bacterial cells through different antimicrobial mechanisms, including attack on the phospholipid bilayer of the cellular membrane, disruption of enzyme systems, damage to the genetic material in the bacteria, and formation of fatty acid hydroperoxidase induced by the oxygenation of unsaturated fatty acids [7].

## 3. Materials and Methods

### 3.1. Plant Material and Isolation of Essential Oils

Pomelo (*Citrus grandis* [L.] Osbeck cv. Matou Wentan) was purchased from the Taiwan Huadong Fruit and Vegetable Producers’ Cooperative. Essential oil was obtained from the fresh peel by using the method described by Fadel et al. with minor modifications [30]. The albedo of fresh pomelo peel was removed, the flavedo layer (1,000 g) was cut into small pieces by using a food processor (Cuisinart DLC-7 Super Pro, Japan), and the essential oil was obtained using a steam distillation device (KC-30, Kou Chou Instrument Co., Ltd, Taipei City, Taiwan) for 3 h. The steam, containing the essential oil, was passed through a cooling system to condense it to form a liquid, and then the essential oil and water were separated. Afterward, the oil was dried over anhydrous sodium sulphate and stored in dark vials at 4 °C until analysis. 

### 3.2. Preparation of Essential Oil Emulsions at Different Homogenizer Speeds

The pomelo peel essential oil obtained through steam distillation was diluted with 1% (*v*/*v*) Tween 20 aqueous solution to 20% (*v*/*v*) essential oil. This mixture was homogenized using a high-speed emulsion homogenizer (Ultra-Turrax T-25; Janke & Kunkel, IKA-Labortechnik, GmbH Co., Staufen, Germany) at 0, 13,500, and 24,000 rpm, for 0.5 min, yielding a 20% (*v*/*v*) essential oil emulsion. A 0.2 μm filter membrane was used for degerming (Gelman Sciences, Ann Arbor, MI, USA), and the essential oil emulsions were diluted with sterile 1% (*v*/*v*) Tween 20 solution to different concentrations to determine antibacterial activity. 

### 3.3. Preparation of Water-Soluble Chitosan

Chitosan (DA = 25%) was purchased from Ohka Enterprises (Kaohsiung City, Taiwan). Water-soluble chitosan was prepared according to the methods described by Mima et al. and Kubota and Eguchi, with minor modifications [31,32]. The chitosan was added to 40% (*w*/*w*) NaOH solution at a ratio of 1:10 and boiled for 4 h. After being washed in deionized water to achieve neutrality, the chitosan was treated again in the alkaline solution for further deacetylation; thus, a highly deacetylated chitosan product was obtained with a 93.0% DA. The deacetylated chitosan product (0.3 g) was dissolved in 30 mL of 1% (*w*/*w*) aqueous acetic acid, mixed with 40 mL of 80% (*w*/*w*) methanol, and the desired amount (0.18 mL) of acetic anhydride was added. After stirring at room temperature for 5 h, 1.0 M, NaOH was added up to pH 8.0 to stop the reaction. Finally, the reaction mixture was dialyzed against deionized water for 3 days and then lyophilized to powder form to determine the degree of *N*-acetylation of the chitosan.

### 3.4. Preparation of Chitosan and Pomelo Peel Essential Oil Emulsion

Sterility control of essential oils and chitosan was performed using the membrane filter method of Owlia et al., with some modifications [33]. To prepare a chitosan and pomelo peel essential oil emulsion, sterilized tryptic soy broth (TSB, Difco Laboratories, Sparks, MD, USA) was added to the prepared water-soluble chitosan on a sterile console, vibrated in a 75 °C water bath for 6 h to dissolution, and filtered by air exhaust through 0.4 μm and 0.2 μm filter membranes in turn, yielding TSB with chitosan at different concentrations (0, 0.01% (*w*/*v*), 0.03% (*w*/*v*), 0.05 (*w*/*v*), and 0.1% (*w*/*v*)). Subsequently, 0.2 mL of an essential oil emulsion (prepared through 24,000 rpm homogenization and 0.2 μm filtration) was added to a spiral test tube with 9.7 mL of TSB. The concentrations of essential oil in the test tube were 0.1% (*v*/*v*), 0.2% (*v*/*v*), and 0.4% (*v*/*v*). Finally, the emulsion was inoculated with 0.1 mL of activated bacteria solution (*S. aureus* or *E. coli*) for antibacterial activity analysis. The control group contained only chitosan (concentrations were 0, 0.01% (*w*/*v*), 0.03% (*w*/*v*), 0.05 (*w*/*v*), and 0.1% (*w*/*v*)), instead of the essential oil emulsion. 

### 3.5. Preparation of Emulsion of Chitosan of Different pH and Essential Oil

To prepare an emulsion of chitosan of different pH and essential oil, 1.0 N HCl and 1.0 N NaOH were added to approximately 30.0 g of TSB, and deionized water was added to reach a volume of 1000 mL; the pH was adjusted to 5.5, 7.4, and 8.5, respectively. The mixtures were sterilized at 121°C for 15 min and added to water-soluble chitosan to prepare TSB with 0.03% (*w*/*v*) chitosan. The preparation method described in Section 3.4 was employed. Afterward, the TSB with chitosan was mixed with 0.2 mL of an essential oil emulsion (prepared through 24,000 rpm homogenization and 0.2 μm filtration), and the essential oil concentration was 0.4% (*v*/*v*). Finally, antibacterial activity analysis was conducted. Samples with only 0.03% (*w*/*v*) water-soluble chitosan or 0.4% (*v*/*v*) essential oil emulsion were used as control groups. In addition, 0.2% (*w*/*v*) water-soluble chitosan was prepared in TSB of different pH values for zeta potential analysis. 

### 3.6. Chemical Analyses

#### 3.6.1. GC–MS

To an essential oil sample of approximately 0.05 g, n-hexane was added to a volume of 5 mL, and GC-MS was used for qualitative analysis of the essential oil composition. The qualitative analysis of the sample was performed using the method described by Deba et al. and Hosni et al., with minor modifications [34,17]. GC-MS analyses were performed on an HP 6890 (II) gas chromatograph interfaced with an HP 5973 mass spectrometer (Agilent Technologies, Palo Alto, CA, USA) with electron impact ionization (70 eV). A DB-5 capillary column (30 m × 0.25 mm, 0.3 μm film thickness, Agilent Technologies, Folsom, CA, USA) was used. The oven temperature was held at 50 °C for 2 min and then increased at 5 °C/min to 250 °C. The carrier gas, helium, was injected at a flow rate of 1.0 mL/min. The scan time and mass range were 1 s and 41–400 *m*/*z*, respectively. The data were recorded as the average of three analyses. Matching of Kovat’s indices [35] and matching of mass spectra were performed for composition identification. The mass spectra used for reference were obtained from the National Institute of Standards and Technology and Wiley databases. 

#### 3.6.2. Gas Chromatography (GC)

To an essential oil sample (0.6 g) or each essential oil standards (0.02 g) (α-pinene, β-pinene, myrcene, limonene, γ-terpinene, *cis*-linalool oxide (f), *cis*-linalool oxide (f) linalool, α-terpineol, neryl acetate and geranyl acetate), n-hexane was added to a volume of 2.0 mL, and 0.8 mL of the essential oil sample solution was mixed with n-hexyl alcohol at an appropriate concentration (n-hexyl alcohol was added to 0.13 g of n-hexane to a volume of 20 mL) to establish an internal standard. The quantitative analysis of each sample was performed using the method described by Hosni et al. and Mohammadi et al., with minor modifications [17,36]. GC analyses were conducted on a Shimadzu GC-14 gas chromatograph (Shimadzu Corporation, Kyoto, Japan), equipped with a flame ionization detector, and a Shimadzu C-R6A chromatopac integraph (Shimadzu Corporation, Kyoto, Japan). An Equity-5 nonpolar column (60 m × 0.25 mm, 0.25 μm film thickness) was used. The column temperature was programmed to rise from 75 to 200 °C at a rate of 2 °C/min. The injector and detector temperature were set at 250 °C. The flow of the carrier gas (N_2_) was 50 mL/min. The injection volume for all samples was 0.5 μL of diluted oil in n-hexane (Merck KGaA, Darmstadt, Germany). The data were recorded as the average of three analyses.

#### 3.6.3. Determination of the Degree of *N*-Acetylation in Water-Soluble Chitosan

The degree of *N*-acetylation in water-soluble chitosan was determined through high-performance liquid chromatography, using the method described by Niola et al. with modifications [37]. A 10 mg sample was placed in a vacuum hydrolysis tube with 0.5 mL of 12 M H_2_SO_4_ and a standard mixture (6.9 mg of oxalic acid dihydrate and 0.25 mL of propionic acid dissolved in 100 mL of deionized water). The tube was closed and placed in a circulating heating bath at 155 °C, and after a 1 h reaction, the tube was placed in ice water for 30 min, and then allowed to reach room temperature. The mixture was completed with 10 mL of deionized water and filtered through a 0.2 μm membrane. Ten microliters of this solution were directly injected into a Synergi C_18_ column (4.6 × 250 mm, particle size, 4 μm; Phenomenex, Torrance, CA, USA) in order to analyze the amount of acetic acid. The sample was analyzed in triplicate. The amount of liberated acetyl groups (mx) was calculated using the following equation:

mx = K×(Ax/Ais) × Mis(1)

where K is the response factor; Ax and Ais are the areas of the acetic and propionic acid peaks, respectively, as the internal standard; and Mis is the amount of the internal standard. The percentage of *N*-acetylation was calculated using the following equation:

degree of *N*-acetylation (%) = (161X/60 − 42X) × 100 (2)

where X = mx/M’; M’ = M-mi (mi = mass of inorganic material including curd protein and ash content); 161 is the mole weight of a 2-amino-2-deoxy-d-glucose unit; and 60 is the mole weight of the acetic acid. 

### 3.7. Zeta Potential Analysis of Chitosan in TSB

The zeta potential in water-soluble chitosan was determined with a Zetasizer (Nano ZSP, Malvern Instruments Corp., Worcestershire, UK), using the method described by Gohtani and Yamano with modifications [38]. The zeta potential measurements were performed in deionized water, which acted as a dispersant, by using a disposable folded capillary cell with the electrodes at 25 °C and an applied voltage of 150 V. The data were recorded as the average of three analyses.

### 3.8. Assays for Antibacterial Activity

#### 3.8.1. Species Culture

The preparation of bacteria was performed using the method described by Friendman et al., with some modifications [39]. *E. coli* (CCRC 10674) and *S. aureus* (CCRC 12652) were purchased from the Bioresource Collection and Research Center of the Food Industry Research and Development Institute (Hsinchu City, Taiwan). The species culture was prepared according to the method described by Carson et al., with some modifications [40]. The bacteria from 1 inoculation loop were cultured in a spiral test tube with TSB at 37 °C for 6 h. The bacterial solution absorbance value was 600 nm, the detected value was 0.33, and the bacterial count was 10^9^ CFU/mL, which was reduced through dilution with TSB to 10^6^ CFU/mL. 

#### 3.8.2. Antibacterial Activity

The antibacterial activity tests were performed using the broth dilution method of Kim and Shin, with some modifications [41]. Samples were prepared before the antibacterial activity was analyzed. Essential oil emulsions and TSB with various chitosan concentrations (0, 0.01% (*w*/*v*), 0.03% (*w*/*v*), 0.05 (*w*/*v*), and 0.1% (*w*/*v*) were prepared according to the previously described procedures. To a spiral test tube with 9.7 mL of TSB, 0.1 mL of the 10^6^ CFU/mL bacterial solution and 0.2 mL of an essential oil emulsion were added; the essential oil concentrations in the tube were 0.4% (*v*/*v*), 0.2% (*v*/*v*), and 0.1% (*v*/*v*). The control group was 1% (*v*/*v*) Tween 20 solution. After 10 h of culture at 37 °C, the samples were diluted with 0.85% (*w*/*v*) NaCl in tryptic soy agar (Difco Laboratories, Sparks, MD, USA) and cultured at 37 °C for 24 h, and the colony counts in various diluted bacterial solution culture dishes were determined (only 30 to 300 colony counts were recorded in the experiments). Each sample was analyzed in triplicate.

#### 3.8.3. Determination of Minimum Inhibitory Concentration

The minimum inhibitory concentration was determined according to the method described by Skandamis et al., with minor modifications [42]. The essential oil emulsions of pomelo peel prepared under different homogenization conditions and *S. aureus* or *E. coli* were cultured at 37 °C for 24 h for antibacterial activity testing. The data were recorded as the average of three analyses. The essential oil concentration and residual bacterial count were plotted to obtain the regression curve, and the minimum essential oil concentration (x) for inhibiting 90% of the bacterial count (y) was obtained according to the following regression equation:

y = ax + b
y: Residual bacterial count (log CFU/mL)
x: Essential oil concentration (%)

### 3.9. Statistical Analysis

Analysis of variance for the results of the aforementioned experiments was performed using Statistical Analysis Systems [43]. Multiple mean comparisons were conducted using Duncan’s multiple range test.

## 4. Conclusions

Use of essential oils and chitosan in the food industry is limited due to their poor solubility in water. In this study, a mixture soluble in a neutral water solution (pH 7.4) was successfully prepared through the homogenization of the essential oil and deacetylated water-soluble chitosan, which have strong synergetic antibacterial effects on food pathogens. The reduction in total bacterial counts of a mixture of a 0.4% pomelo peel essential oil emulsion and 0.03% water-soluble chitosan in a neutral water solution on *S. aureus* was higher than that of the 0.4% essential oil emulsion or 0.03% water-soluble chitosan alone by approximately 1.8 and 4.6 times, respectively, and, for *E. coli*, by approximately 2.2 and 4.8 times, respectively. The composition of the essential oil of Taiwan Matou pomelo peel, obtained through steam distillation, was analyzed through GC-MS. A total of 33 compounds were identified, and the main constituent was limonene (940.07 mg/g). The results showed that a pomelo peel essential oil emulsion homogenized at 24,000 rpm had a stronger inhibitory effect on *S. aureus* and *E. coli* than unhomogenized essential oil, with the MIC value being 1.9 times lower. Therefore, this study suggests using a mixture of emulsified pomelo peel oil and water-soluble chitosan to develop a novel natural food preservative, which could prevent the growth of pathogens in foods and extend their shelf life. Additionally, this study indicates that the processability of food, as well as the economic value of the byproducts of the Taiwan Matou pomelo and chitosan, could be increased.

## Figures and Tables

**Table 1:** Constituents of pomelo peel oil isolated by steam distillation.

Peak No.	Constituents	K.I.^a^	Concentration	
			Relative(%)	Absolute (mg/g)
1	α-pinene	938	0.8	4.66
2	β-pinene	980	2.7	13.53
3	myrcene	991	3.1	23.65
4	α-phellandrene	1002	0.1	
5	limonene	1031	87.5	940.07
6	*trans*-β-ocimene	1048	0.4	
7	γ-terpinene	1061	0.1	0.24
8	*cis*-linalool oxide (f)	1072	0.6	3.82
9	α-terpinolene	1086	0.1	
10	*trans*-linalool oxide (f)	1089	0.3	2.15
11	linalool	1098	0.4	2.48
12	*trans*-*p*-2,8-mentha-dien-1-ol	1121	0.0	
13	*cis*-limonene oxide	1135	0.0	
14	*trans*-limonene oxide	1140	0.0	
15	β-terpineol	1147	-	
16	terpinen-4-ol	1176	0.1	
17	α-terpineol	1188	0.3	2.18
18	*trans*-carveol	1216	0.0	
19	nerol	1228	0.1	
20	*cis*-carveol	1229	0.1	
21	neral	1237	0.1	
22	carvone	1243	0.0	
23	geraniol	1250	0.1	
24	geranial	1266	0.2	
25	neryl acetate	1363	0.0	0.072
26	geranyl acetate	1380	0.0	0.25
27	β-elemene	1390	0.0	
28	α-cedrene	1410	0.2	
29	β-caryophyllene	1420	0.2	
30	germacrene-d	1486	0.4	
31	*δ*-cadinene	1523	0.0	
32	(E)-nerolidol	1561	0.0	
33	caryophyllene oxide	1582	0.0	

^a^ Relative Kovat’s indices, experimental: n-alkanes (C9–C24) were used as reference points to calculate relative retention indices.

**Table 2:** Effect of homogenization speed and concentration of emulsified pomelo peel oil on the viability of *S. aureus* and *E. coli* (Log CFU/mL) ^a,b^.

Bacteria	rpm	Concentration of Essential Oil (%)			
		0^c^	0.1	0.2	0.4
*S. aureus*	0	9.00 ± 0.14 ^Ax^	8.32 ± 0.07 ^Bx^	8.07 ± 0.11 ^BCx^	7.97 ± 0.23 ^Cx^
	13,500	8.97 ± 0.06 ^Ax^	8.21 ± 0.09 ^Bx^	8.16 ± 0.02 ^BCx^	8.07 ± 0.07 ^Cx^
	24,000	8.97 ± 0.06 ^Ax^	8.20 ± 0.03 ^Bx^	8.05 ± 0.11 ^Cx^	7.81 ± 0.01 ^Dx^
*E. coli*	0	9.03 ± 0.07 ^Ax^	8.44 ± 0.15 ^Bx^	8.25 ± 0.03 ^BCx^	8.18 ± 0.13 ^Cx^
	13,500	8.95 ± 0.15 ^Ax^	8.49 ± 0.03 ^Bx^	8.26 ± 0.14 ^Cx^	7.85 ± 0.06 ^Dy^
	24,000	8.95 ± 0.15 ^Ax^	8.38 ± 0.06 ^Bx^	7.91 ± 0.12 ^Cy^	7.72 ± 0.03 ^Cz^

^a^ Means within a row with different superscripts (A–D) differ significantly (*p* < 0.05), n = 3. ^b^ Means within a column with different superscripts (x–z) differ significantly (*p* < 0.05), n = 3. ^c^ 1% Tween 20 solution without homogenization.

**Table 3:** Effect of chitosan concentration on the viability of *S. aureus* and *E. coli* (Log CFU/mL) ^a,b^.

Bacteria	Concentration of Chitosan (%)				
	Control	0.01	0.03	0.05	0.1
*S. aureus*	9.05 ± 0.04 ^Ax^	8.56 ± 0.03 ^Bx^	8.53 ± 0.02 ^Bx^	7.93 ± 0.16 ^Cx^	8.09 ± 0.15 ^Cx^
*E. coli*	9.01 ± 0.01 ^Ax^	8.57 ± 0.04 ^Bx^	8.54 ± 0.01 ^Bx^	8.18 ± 0.07 ^Cx^	7.94 ± 0.05 ^Dx^

^a^ Means within a row with different superscripts (A–D) differ significantly (*p* < 0.05), n = 3. ^b^ Means within a column with different superscripts (x) differ significantly (*p* < 0.05), n = 3.

**Table 4:** Effect of chitosan concentration in 0.4% emulsified pomelo peel oil on the viability of *S. aureus* and *E. coli* (Log CFU/mL) ^a,b^.

Bacteria	Concentration of Chitosan (%)					
	Control	0	0.01	0.03	0.05	0.1
*S. aureus*	9.16 ± 0.09 ^Ax^	8.10 ± 0.07 ^Bx^	7.19 ± 0.22 ^Cx^	6.76 ± 0.17 ^Dx^	6.82 ± 0.13 ^Dx^	6.99 ± 0.26 ^CDx^
*E. coli*	9.18 ± 0.01 ^Ax^	7.93 ± 0.01 ^By^	7.39 ± 0.15 ^Cx^	6.92 ± 0.26 ^Dx^	6.63 ± 0.06 ^Ex^	7.10 ± 0.16 ^DEx^

^a^ Means within a row with different superscripts (A–E) differ significantly (*p* < 0.05), n = 3. ^b^ Means within a column with different superscripts (x–y) differ significantly (*p* < 0.05), n = 3.

**Table 5:** Minimum inhibitory concentration (MIC) of emulsified pomelo peel oil and chitosan against *S. aureus* and *E. coli*.

Bacteria	MIC				
	Essential Oil (%)			Chitosan (%)	
	0 (rpm)	13,500 (rpm)	24,000 (rpm)	+0% E.O.	+0.4% E.O.
*S. aureus*	0.29	>0.29	0.27	0.046	0.019
*E. coli*	0.49	0.34	0.25	0.086	0.034

**Table 6:** Effects of adding emulsified pomelo peel oil, chitosan, and E.O.-chitosan in broths of different pH values on the inhibitory percentage (%) of *S. aureus* and *E. coli* ^a,b^.

Bacteria	Sample	pH		
		5.5 ^c^	7.4 ^d^	8.5 ^e^
*S. aureus*	0.4% E.O.	81.20 ± 2.22 ^Cx^	90.04 ± 1.04 ^By^	95.10 ± 0.65 ^Ax^
	0.03% chitosan	85.47 ± 1.33 ^Ax^	65.26 ± 2.09 ^Bz^	61.83 ± 1.90 ^By^
	0.4% E.O. + 0.03% chitosan	82.58 ± 0.77 ^Cx^	99.48 ± 0.25 ^Ax^	97.46 ± 0.94 ^Bx^
*E. coli*	0.4% E.O.	78.11 ± 0.40 ^By^	92.70 ± 0.62 ^Ay^	71.29 ± 2.56 ^Cy^
	0.03% chitosan	76.04 ± 1.63 ^Ay^	62.61 ± 3.70 ^Bz^	66.06 ± 6.71 ^ABy^
	0.4% E.O. + 0.03% chitosan	91.68 ± 4.56 ^Bx^	99.06 ± 0.07 ^Ax^	95.06 ± 0.63 ^ABx^

^a^ Means within a row with different superscripts (A–C) differ significantly (*p* < 0.05), n = 3. ^b^ Means within a column with different superscripts (x–z) differ significantly (*p* < 0.05), n = 3. ^c^ Zeta potential of chitosan is 5.4 mV. ^d^ Zeta potential of chitosan is −3.8 mV. ^e^ Zeta potential of chitosan is −3.2 mV.
